# Long-Term Conservation Agriculture Improves Soil Quality in Sloped Farmland Planting Systems

**DOI:** 10.3390/plants13233420

**Published:** 2024-12-05

**Authors:** Hongying Li, Jun Tang, Jing Wang, Jun Qiao, Ningyuan Zhu

**Affiliations:** 1 State Key Laboratory of Soil and Sustainable Agriculture, Institute of Soil Sciences, Chinese Academy of Sciences, No. 298 Chuangyou Road, Nanjing 211135, China; tangjun@issas.ac.cn (J.T.); jqiao@issas.ac.cn (J.Q.); 2 Robert R. McCormick School of Engineering and Applied Science, Northwestern University, 633 Clark Street, Evanston, IL 60208, USA; jingwang2024@u.northwestern.edu; 3 College of Environment, Hohai University, Nanjing 210098, China; zhuny0106@hhu.edu.cn

**Keywords:** soil erosion, orchard, crop field, discriminant analysis, soil quality index

## Abstract

Conservation agriculture practices (CAs) are important under the increasingly serious soil quality degradation of sloping farmlands worldwide. However, little is known about how the long-term application of CAs influences soil quality at different slope positions. We conducted field experiments for a watershed sloping farmland’s mainstream planting systems in the Three Gorges Reservoir area of China. Orchard plots were treated with a conventional citrus planting pattern (C-CK), citrus intercropped with white clover (WC), citrus orchard soil mulched with straw (SM) and citrus intercropped with *Hemerocallis flava* contour hedgerows (HF). Crop field plots were treated with a conventional wheat–peanut rotation (W-CK), a wheat–peanut rotation intercropped with *Toona sinensis* contour hedgerows (TS), a wheat–peanut rotation intercropped with alfalfa contour hedgerows (AF) and a ryegrass–sesame rotation (RS). We collected soil samples from the plots at the upper, middle and lower slope positions and measured their soil properties after a nine-year experiment. We found that (1) CAs improved the soil properties at the three slope positions; (2) the effect of the CAs on the soil properties was more significant than that on the slope position; and (3) the soil quality index at the upper, middle and lower slope positions increased by 29.9%, 45.8% and 33.3%, respectively, for WC; 48.7%, 39.5% and 27.1%, respectively, for SM; and 21.7%, 25.5% and 21.6%, respectively, for HF compared to C-CK; as well as 18.7%, 23.7% and 20.4%, respectively, for TS; 16.9%, 18.6% and 16.5%, respectively, for AF; and 16.1%, 13.0% and 13.9%, respectively, for RS compared to W-CK. These findings suggest that long-term CA application enhances the soil quality of the slope position, of which SM and TS applied to orchards and crop fields, respectively, are the most effective.

## 1. Introduction

Sloping farmland constitutes an important part of cultivated land resources and plays a vital role in ensuring global food security [1,2]. At present, the greatest problem in the utilization of sloping farmland is severe soil erosion [3]. Soil erosion leads to the soil quality degradation of sloping farmland by destroying the soil structure [4], thinning the plow layer, reducing the organic matter and nutrient contents and deteriorating the downstream water quality [5,6]. These issues pose a threat not only to global soil productivity and sustainable agricultural development but also to water ecological environment security [7]. Therefore, the soil quality degradation of sloping farmland caused by soil erosion during sloping farmland utilization has become a hot topic of worldwide concern [8].

Conservation agriculture practices (CAs) constitute the best way to solve the soil quality degradation of sloping farmland. CAs are a series of practices applied to agricultural production, which protect the environment from negative influences by agricultural production activities and aid in balancing efficient production and protecting the environment [9,10], for example, soil surface mulching [11,12], adjustments to agricultural planting patterns [13], contour hedgerows [14], silt dam systems [15] and the conversion of sloping farmland to terraces [16]. CAs can sustain soil quality [17,18] and mitigate agricultural nonpoint source pollution [19] by preventing runoff from causing erosion and nutrient losses and allowing for water to infiltrate the ground in sloping farmland [20,21].

Previous studies on the influences of CAs on soil properties have focused on mostly the short-term influences of single CAs on a few soil physicochemical properties. Studies conducted in Old Goa in Goa, India, have suggested that contour trenches with vegetative barriers significantly increase the soil organic carbon stocks of sloping farmland compared with controls [22]. Contour hedgerows slow the rate of runoff and create backwater strips in front of hedgerows; as a result, sediment is predominantly deposited near hedgerows [23]. Therefore, these CAs can reduce the soil bulk density, maintain soil fertility and increase the porosity and water infiltration rate of the cultivated soil [24,25]. However, little is known about how the long-term application of multiple CAs influences soil quality at different slope positions of sloping farmland through more comprehensive soil properties, whether CAs or the slope position has a greater influence on the soil properties and which CAs are the most effective. Such findings will be crucial in guiding decision-makers and farmers on how to employ more effective CAs to achieve the sustainable utilization of sloped farmland [26]. Thus, it is very important to study the effects of the long-term application of CAs on comprehensive soil properties and soil quality at different slope positions of sloping farmland for mainstream planting systems in watersheds.

The TGRA of China is a key sensitive ecological region [27] with large areas of sloping farmland. This region has one of the most serious areas of soil erosion in China because of its frequent high-energy rainstorms, loose soil structure and severely eroded topographic conditions [28]. The quality of the agricultural environment in the TGRA is related not only to the sustainable agricultural development of this area and the safety of the Three Gorges Dam [29] but also to the ecological health and water security of the middle and lower reaches of the Yangtze River [30]. Hence, we conducted a nine-year field experiment of multiple CAs for orchards and crop fields in two mainstream planting systems of sloping farmland in the TGRA. We collected and measured soil samples at the upper, middle and lower slope positions of sloping farmland after 9 years. Our objectives were to (1) clarify the influences of different CAs on the soil physicochemical and microbial properties at the different slope positions of sloping farmlands in orchards and crop fields; (2) discriminate the magnitudes of the influences of the CAs and the slope position on the soil properties of sloping farmlands in orchards and crop fields separately; (3) evaluate the variations in the soil quality at the different slope positions of the sloping farmlands after the long-term application of CAs; and (4) identify the optimal CAs for the sloping farmlands in orchards and crop fields. This study is essential to clarify the mechanism for controlling the soil quality degradation of watershed sloping farmland by CAs and to find the optimal CAs for watershed sloping farmland use to ensure the sustainable utilization of sloping farmland worldwide.

## 2. Results and Discussion

### 2.1. Influence of the CAs on the Soil Physical Properties at Different Slope Positions

**Figure 1:**
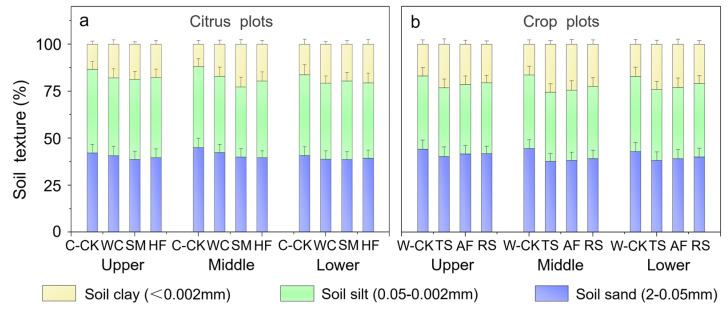
Soil sand, silt and clay contents at the different slope positions in the citrus experimental plots (**a**) and the crop experimental plots (**b**). The error bars represent the standard deviation (*n* = 3).

After 9 years of field experiments, the soil physical properties at different slope positions were significantly influenced by the application of the CAs (Figure 1). In the citrus experimental plots, compared with that in the C-CK plots, the soil clay content at the upper, middle and lower slope positions increased by 34.2%, 43.6% and 28.3%, respectively, for WC; by 40.7%, 91.5% and 20.9%, respectively, for SM; and by 32.9%, 65.0% and 26.7%, respectively, for HF. However, the soil silt-sand content decreased by 5.3%, 5.9% and 5.5%, respectively, for WC; by 6.3%, 12.3% and 4.0%, respectively, for SM; and by 5.1%, 8.8% and 5.2%, respectively, for HF (Figure 1a). In the crop experimental plots, compared with that in the W-CK plots, the soil clay content at the upper, middle and lower slope positions increased by 37.3%, 55.0% and 41.6%, respectively, for TS; by 26.5%, 48.6% and 34.7%, respectively, for AF; and by 21.8%, 36.9% and 22.0%, respectively, for RS. However, the soil silt-sand content decreased by 7.6%, 10.8% and 8.6%, respectively, for TS; by 5.4%, 9.6% and 7.1%, respectively, for AF; and by 4.4%, 7.3% and 4.5%, respectively, for RS (Figure 1b).

The long-term application of the CAs reduced soil clay loss, resulting in an improvement in the soil mechanical composition at the three slope positions in the CA-treated plots because the CAs increased ground cover, reduced runoff velocity and flow and intercepted sediment. The greatest increase in the soil clay content and reduction in the soil silt-sand content in the CA-treated plots were observed at the middle slope position. This was mainly because this position is most strongly affected by the erosion of rainwater runoff on the slope [31], and the application of CAs effectively reduced the effects of runoff scouring. The differences in the improvement in soil texture at the three slope positions among the CA-treated plots revealed that these CAs have different control abilities for soil loss.

The results revealed that (1) the CAs improved the soil texture at each slope position; (2) the increases in the soil clay content at the middle slope position were the greatest among the three slope positions; (3) SM was the best, followed by WC and HF, in terms of improving the soil texture at the three slope positions overall in the citrus experimental plots; and (4) TS was the best, followed by AF and RS, in terms of improving the soil texture at the three slope positions overall in the crop experimental plots. The findings of our study and those of previous studies [32] indicate that a hedgerow system can increase the fine particle content of the soil tillage layer.

### 2.2. Influence of the CAs on the Soil Chemical Properties at the Different Slope Positions

**Figure 2:**
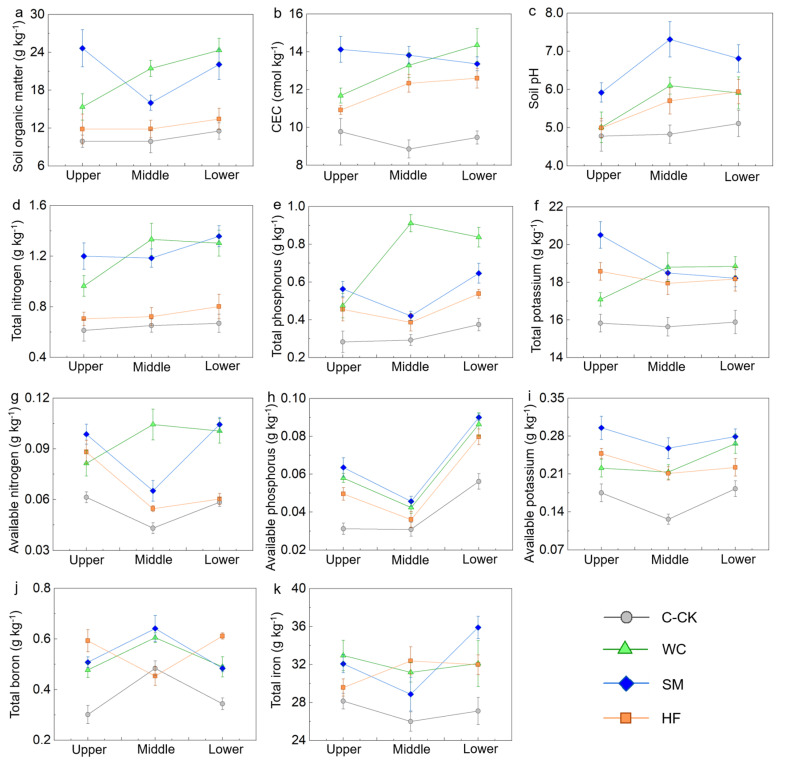
Soil chemical properties at the different slope positions in the citrus experimental plots. The error bars represent the standard deviation (*n* = 3). (**a**) Soil organic matter; (**b**) CEC; (**c**) soil pH; (**d**) total nitrogen; (**e**) total phosphorus; (**f**) total potassium; (**g**) available nitrogen; (**h**) available phosphorus; (**i**) available potassium; (**j**) total boron; (**k**) total iron.

**Figure 3:**
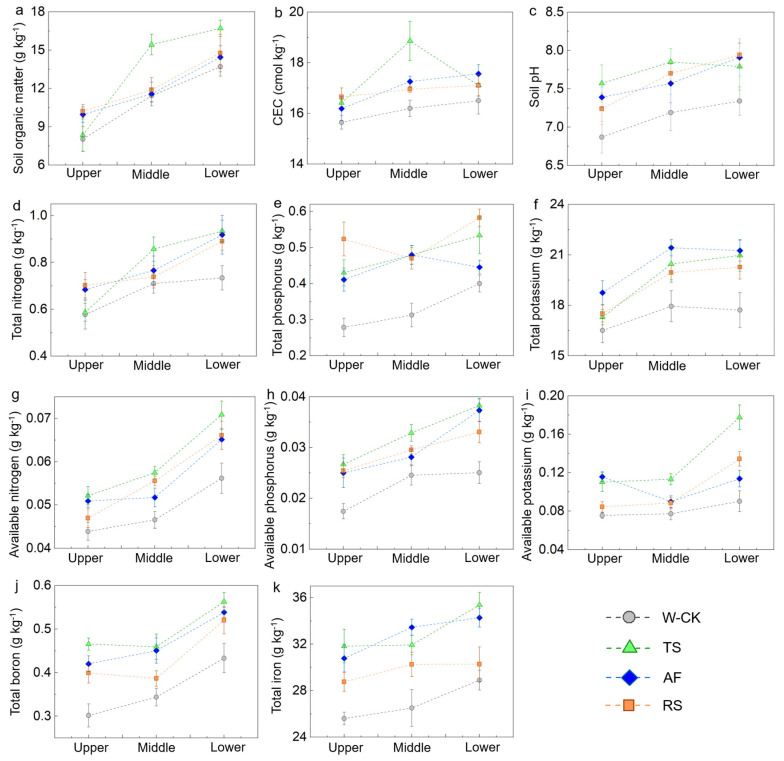
Soil chemical properties at the different slope positions in the crop experimental plots. The error bars represent the standard deviation (*n* = 3). (**a**) Soil organic matter; (**b**) CEC; (**c**) soil pH; (**d**) total nitrogen; (**e**) total phosphorus; (**f**) total potassium; (**g**) available nitrogen; (**h**) available phosphorus; (**i**) available potassium; (**j**) total boron; (**k**) total iron.

Compared with those in the C-CK and W-CK plots, the soil chemical properties increased at different slope positions in the CA-treated plots, except for the total boron (TB) content at the middle slope position in the HF (Figure 2 and Figure 3). In the citrus experimental plots, six properties at two or more slope positions and five properties at one slope position in SM had the highest values in all the treatments; three properties at two or more slope positions and four properties at one slope position in WC had the highest values in all the treatments; and one property at two slope positions in HF had the greatest value in all the treatments (Figure 2). 

The increases in the soil clay content at each slope position after the application of the CAs improved the soil capacity to combine nutrients [33,34]. Furthermore, the decomposition of straw, white clover litter and *Hemerocallis flava* litter increased the soil organic matter and nutrient contents [35,36,37]. White clover can fix atmospheric N [38], which provides a very large amount of nitrogen input to the soil and has a significant influence on the balance and turnover of nitrogen in the ecosystem [39]. Organic matter is vital for soil cation binding and acid buffering, and soil clay is capable of absorbing significant amounts of exchangeable ions due to its large surface area [40,41]. The results of the correlation analysis also revealed that the soil cation exchange capacity (CEC) and pH were significantly correlated with the soil organic matter (SOM) content and the soil clay content in each treatment (*p* < 0.05). The differences in the improvement in the soil chemical properties at the three slope positions among the CA-treated plots revealed that these CAs have different abilities to reduce soil clay and nutrient losses and increase the soil organic matter and nutrient contents. The results demonstrated that (1) these CAs were effective at improving the soil chemical properties at each slope position, and (2) SM had the greatest effect, followed by WC and HF in general, in the citrus orchards.

In the crop experimental plots, eight properties at two or more slope positions and one property at one slope position in the TS had the highest values in all the treatments; one property at three slope positions and four properties at one slope position in the AF had the highest values in all the treatments; and one property at two slope positions and four properties at one slope position in the RS had the highest values in all the treatments (Figure 3).

Contour hedgerows effectively reduce soil clay loss and increase SOM input through root turnover [34,42]. Ryegrass and sesame reduced soil clay loss by providing good vegetation cover and fixing soil, which subsequently reduced sediment erosion and nutrient losses [43]. The soil CEC and acid buffering capacity increased because the CAs effectively intercepted the soil clay and increased the SOM content [44,45,46]. The differences in the improvement in soil chemical properties at the three slope positions among the CA-treated plots revealed that these CAs have different abilities to reduce soil clay and nutrient losses and increase soil organic matter. The results demonstrated that (1) these CAs were effective at improving soil chemical properties at each slope position, and (2) TS had the greatest effect, followed by AF and RS, in the crop fields.

### 2.3. Influence of the CAs on the Soil Microbial Properties at the Different Slope Positions

Compared with those of the C-CK and W-CK plots, the soil microbial properties increased at different slope positions in the CA-treated plots, except for hydrogen peroxidase (HP), urease (Ur) and alkaline phosphatase (ALP) at one slope position in WC, HP and SE at one slope position in SM, HP at one slope position in HF, and HP and ALP at one slope position in RS (Table 1). In the citrus experimental plots, four soil microbial properties at two or more slope positions and three soil microbial properties at one slope position in WC presented the highest values in all the treatments; three microbial properties at two slope positions and two soil biological properties at one slope position in SM presented the highest values in all the treatments; and one soil microbial property at two slope positions and two soil microbial properties at one slope position in HF presented the highest values in all the treatments (Table 1). The results revealed that (1) these CAs improved the soil microbial properties at each slope position in general, and (2) WC had the greatest effect, followed by SM and HF, in general, in the sloping farmlands of citrus orchards.

In the crop experimental plots, five soil microbial properties at two or more slope positions and one soil microbial property at one slope position in TS presented the highest values among all the treatments; three microbial properties at two slope positions and two soil microbial properties at one slope position in AF presented the highest values among all the treatments; and two soil microbial properties at one slope position in RS presented the highest values among all the treatments (Table 1). The results show that (1) these CAs were highly effective at improving soil microbial properties at each slope position in general, and (2) TS had the greatest effect, followed by AF and RS in general, in the sloping farmland of crop fields.

The long-term application of CAs resulted in a significant increase in the contents of organic matter, nitrogen, phosphorus and other nutrients in the soil, which are sources of energy and nutrients for soil microorganisms [47,48] and improve soil ecology [49]. Therefore, the CAs enhanced the soil microbial biomass and enzyme activities at all slope positions. The differences in the improvement in the soil microbial properties at the three slope positions among the CA-treated plots revealed that these CAs have different abilities to increase the contents of organic matter, nitrogen, phosphorus and other nutrients in the soil and improve the soil ecology. Previous studies have also confirmed that an increase in the soil content of organic matter and nutrients increases the soil microbial biomass and enzyme activities [36,50].

### 2.4. Discriminant Analysis of Soil Properties

**Figure 4:**
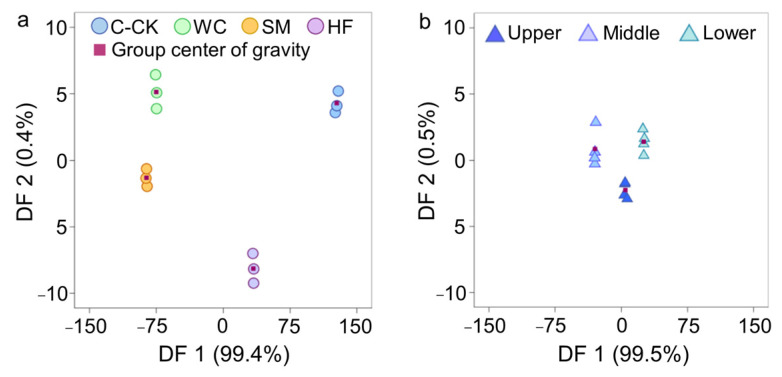
The joint distribution map of discriminant function 1 (DF1) and discriminant function 2 (DF2). DF1 and DF2 in (**a**,**b**) were generated based on the soil properties of the C-CK, WC, SM and HF treatments and the soil properties at the different slope positions using DA in the citrus experimental plots, respectively.

In the citrus experimental plots, three DFs (DF1, DF2 and DF3) were generated based on the soil properties of the C-CK, WC, SM and HF treatments using DA, and all DFs were significant (*p* < 0.01) (Table 2). Two DFs (DF1 and DF2) were generated based on the soil properties at the upper, middle and lower slope positions using DA, and only DF1 was significant (*p* < 0.01). The joint distribution maps of DF1 and DF2 revealed that the distance of the group gravity center between the WC, SM and HF and the C-CK was far greater than that among the upper, middle and lower slope positions (Figure 4a,b). The clear separation of soil properties and the relatively high eigenvalues of the DFs revealed that CAs have a more significant impact on soil properties than does slope position in the sloping farmland of citrus orchards. In addition, the distance of the group gravity center between the WC, SM and HF and the C-CK indicated that the SM had the greatest effect, followed by the WC and HF, in improving the soil properties of the sloping farmland in citrus orchards.

In the crop experimental plots, one DF (DF1) was generated based on the soil properties of the W-CK, TS, AF and RS treatments using DA, and DF1 was significant (*p* < 0.01) (Table 2). One DF (DF1) was generated based on the soil properties at the upper, middle and lower slope positions using DA, and DF1 was significant (*p* < 0.01). The eigenvalues of DF1 from the four treatments were significantly greater than that of DF1 from the three slope positions. The results indicated that the CAs have a more significant effect on the soil properties than does the slope position in the sloping farmland in crop fields.

As noted above, the CAs had a greater effect on the soil properties than did the slope position in the sloping farmland. That is, the CAs effectively controlled soil erosion on sloping farmland and reduced the variances in soil properties across slope positions, which is highly conducive to the sustainable use of sloping farmland. The effects of the different CAs on the soil properties also differed. These results occurred because (1) the CAs reduced the soil erosion of sloping farmland, and (2) the magnitudes of the reduction in the soil erosion of sloping farmland among the CAs were different. Our study confirms the conclusions of Liu et al. (2018) [45], who indicated that discriminant analysis can be used to effectively discriminate soil properties measured routinely at different levels.

### 2.5. Soil Quality Responses to the Long-Term Application of CAs

**Figure 5:**
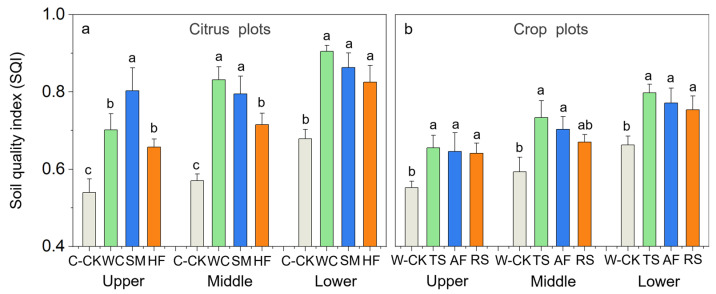
The soil quality index (SQI) values at different slope positions and under different treatments in the citrus experimental plots (**a**) and the crop experimental plots (**b**). The error bars represent the standard deviation (*n* = 3). Different letters indicate significant differences at *p* < 0.05.

The soil quality at the upper, middle and lower slope positions in the sloping farmlands of the citrus orchards and crop fields was quantitatively evaluated using the soil quality index (SQI). The SQI values increased significantly at each slope position in the CA-treated plots (Figure 5). Compared with C-CK, the SQI values at the upper, middle and lower slope positions increased by 29.9%, 45.8% and 33.3%, respectively, for WC; by 48.7%, 39.5% and 27.1%, respectively, for SM; and by 21.7%, 25.5% and 21.6%, respectively, for HF (Figure 5a). Compared with W-CK, the SQI values at the upper, middle and lower slope positions increased by 18.7%, 23.7% and 20.4%, respectively, for TS; by 16.9%, 18.6% and 16.5%, respectively, for AF; and by 16.1%, 13.0% and 13.9%, respectively, for RS (Figure 5b).

These results occurred because the long-term application of these CAs significantly improved the soil physicochemical and microbial properties at the upper, middle and lower slope positions in the sloping farmlands of citrus orchards and crop fields, and differences existed among these CAs in improving the soil properties. The results revealed that (1) these CAs effectively enhance the soil quality at each slope position in the sloping farmlands of citrus orchards and crop fields; (2) the effects of SM were the greatest, followed by those of WC and HF in the sloping farmlands of citrus orchards; and (3) the effects of TS were the greatest, followed by those of AF and RS in the sloping farmlands of crop fields.

## 3. Materials and Methods

### 3.1. Study Site

The study site is located in the head region of the TGRA (31°4′ N, 110°41′ E, 224 m). This region has a humid subtropical monsoon climate, characterized by an average annual temperature and rainfall of 22.1 °C and 1114.9 mm, respectively [51]. The soil type for sloping farmland is predominantly purple soil (inceptisol) [52]. The mainstream planting systems of sloping farmland are citrus orchards in valley areas below 600 m above sea level and crop fields in highlands above 600 m above sea level, respectively.

### 3.2. Experimental Design

**Figure 6:**
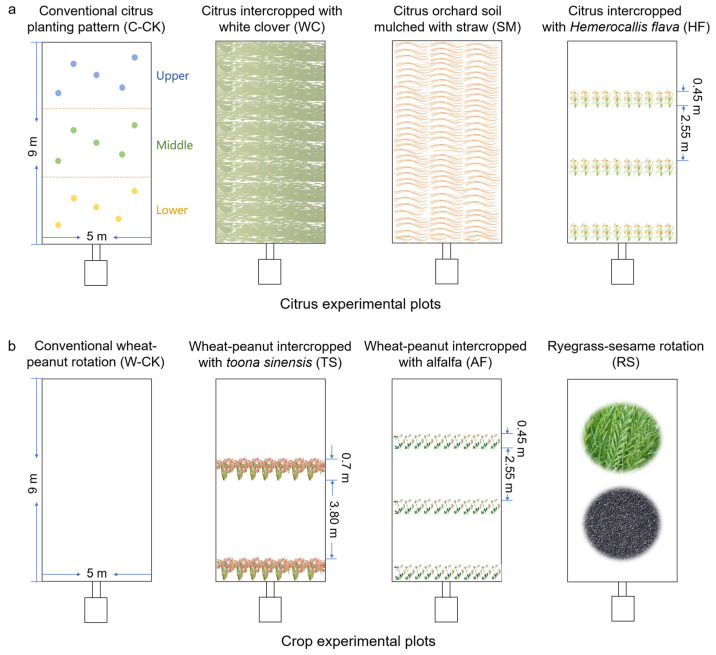
Diagrams of the experimental plots (**a**,**b**) and the soil sampling points (**a**) (the sampling sites of the other plots were the same as those shown in (**a**)).

The field experiment consisted of eight experimental plots, four of which were planted for citrus, whereas the others were planted for crops (Figure 6). Each plot was designed as a 9 m × 5 m area and was surrounded by waterproof walls. The slope of each plot was 25°, which is a typical slope of local farmland. At the bottom of each plot was a runoff pool, which was connected to the others. A protection belt was established between the plots.

Four treatments were established in the citrus experimental plots, as shown in Figure 6a. White clover was sown in WC only once before the experiment began (7.5 kg ha^−1^) because it is a nitrogen-fixing perennial herb. The annual application amount of wheat straw in SM was 7.3 t ha^−1^. Compound fertilizer was applied by furrow application under the citrus trees (Table 3).

Four treatments were established for the crop experimental plots, as shown in Figure 6b. The annual ryegrass and sesame seeding rates were 6.0 kg ha^−1^ and 15.0 kg ha^−1^, respectively. The compound fertilizer was spread on the field and then plowed into the soil before sowing the crops (Table 3).

The conditions of all the treatments were the same before the experiment. The experiment was initiated in 2011 and lasted for 9 years (2011–2019).

### 3.3. Sample Collection and Analysis

Five sampling points, arranged in an S-shaped pattern, were selected for soil sampling at each slope position (Figure 6a). The soil samples (0–20 cm) were collected from each sampling point in April 2020, and all of the soil samples from each slope position were mixed thoroughly to form a single composite sample.

Some of each composite soil sample was air-dried and passed through a 2 mm sieve to determine the physiochemical properties. Another portion of each of these composite soil samples was stored at 4 °C for determination of the soil enzyme activity and soil microbial biomass. The methods for determining soil properties except available nitrogen (AN) are shown in Table 4. AN was considered equal to the sum of NH_4_^+^-N and NO_3_^−^-N [53]. NH_4_^+^-N and NO_3_^−^-N were extracted using indophenol blue-KCl and KCl, respectively, and the colorimetric method was used to determine their contents in solution [54].

### 3.4. Discriminant Analysis

Discriminant analysis (DA) has been successfully used in soil property analysis [64]. DA is a multivariate statistical analysis method for classifying and identifying observed quantitative characteristics (called factors or discriminant variables) [65]. The DA stepwise method was used in this study to distinguish the effects of the slope positions and the treatments on the soil properties. First, the discriminant functions (DFs) of the sloping farmlands in citrus orchards and crop fields were established based on the soil properties observed in different treatments. The soil properties included the 22 soil physicochemical and microbial properties, which were measured as described in Section 3.3. Second, the classification criteria for the sloping farmland in citrus orchards and crop fields were established by dividing the soil properties into four groups and three groups according to the treatment number (four treatments) and the slope position number (three slope positions) in the sloping farmland in citrus orchards and crop fields, respectively. The established DFs were used to calculate eigenvalues, which were then used as measures of the classification. The larger the eigenvalue of the DF, the stronger the ability to distinguish the DF categories.

### 3.5. Soil Quality Evaluation

#### 3.5.1. Selection of Evaluation Factors

A total dataset composed of 22 evaluation factors was selected for soil quality evaluation, including three soil physical properties (soil sand, silt and clay), eleven soil chemical properties (SOM, CEC, pH, TN, TP, TK, AN, AP, AK, TFe and TB) and eight soil microbial properties (MBC, MBN, MBP, ALP, ACP, Ur, HP and SE).

#### 3.5.2. Single-Factor Evaluation

Among the evaluation factors, the first category had critical values, and they were evaluated using formula (1), which included SOM, CEC, TN, TP, TK, AN, AP, AK, MBC, MBN, MBP, ALP, ACP, Ur, HP and SE [66]. The other category had optimal critical ranges, and they were evaluated using formula (2), which included soil sand, silt, clay, pH, TFe and TB.

(1) $$S\left(x\right)=\left\{\;\;\;\;\begin{array}{c} 1\;\;\;\;\;\;\;\;\;\;x\geq x_{0}\;\\ \frac{x}{x_{0}}\;\;\;\;\;\;\;x\leq x_{0}\; \end{array}\right.$$

(2) $$S\left(x\right)=\left\{\begin{array}{c} \;\;1\;\;\;\;\;\;\;\;\;\;\;\;\;\;\;\;\;\;\;\;\;\;\;\;\;\;\;\;\;\;b_{1}\leq x\leq b_{2}\\ \frac{x-a_{1}}{b_{1}-a_{1}}\;\;\;\;\;\;\;\;\;\;\;\;\;\;\;\;\;\;\;\;\;\;\;a_{1}\leq x\leq b_{1}\\ \frac{a_{2}-x}{\;a_{2}-b_{2}}\;\;\;\;\;\;\;\;\;\;\;\;\;\;\;\;\;\;\;\;\;\;\;b_{2}\leq x\leq a_{2}\\ \;\;\;\;\;\;\;0\;\;\;\;\;\;\;\;\;\;\;\;\;\;\;\;\;\;\;\;\;\;\;\;\;x\leq a_{1}\;or\;x\geq a_{2} \end{array}\right.$$

Here, *x* and *S*(*x*) are the value and the score function of the evaluation factor; and *x*_0_, *a*_1_, *a*_2_, *b*_1_ and *b*_2_ are the critical values of the evaluation factor determined according to the actual situation.

#### 3.5.3. Calculation of the Weights of the Evaluation Factors

First, the principal component analysis (PCA) method was used to calculate the communality of each evaluation factor [67]. The weight of each factor was subsequently determined by dividing its communality by the sum of the commonality of all factors [68,69].

#### 3.5.4. Calculation of the SQI

The soil quality index (SQI) is a method used to quantitatively evaluate soil quality, which has been used by many scholars and has obtained satisfactory evaluation results [70,71,72]. The SQI values were calculated using the following formula [73]:

(3) $$\mathrm{SQI}=\sum_{i=1}^{n}W_{i}S_{i}$$

Here, *i* and *n* are the evaluation factors and their number, and *W_i_* and *S_i_* are the evaluation factor weights and scores, respectively.

### 3.6. Statistical Analysis

The effects of various treatments and slope positions on soil properties were distinguished using DA. The relationships between the soil properties were analyzed by correlation analysis, with the least significant differences at the 5% level. The weights of the soil quality evaluation factors were calculated by PCA. The differences in the soil microbial properties and SQI among the different treatments in the citrus plots and crop plots were tested using analysis of variance (ANOVA) based on the least significant difference at the 5% level. All the statistical analyses were carried out using SPSS (version 23, SPSS, Inc., Chicago, IL, USA).

## 4. Conclusions

Based on a nine-year field experiment for the mainstream planting systems of watershed sloping farmland, our results demonstrate that (1) the long-term application of CAs improves the soil properties and soil quality at the upper, middle and lower slope positions in the sloping farmlands of citrus orchards and crop fields; (2) CAs have a more significant effect on soil properties than do the slope positions; and (3) SM and TS are the best at enhancing the soil quality of the sloping farmlands in citrus orchards and crop fields, respectively. Future research should focus on exploring the effects of CAs on the yield following improvements in soil quality.

## Figures and Tables

**Table 1:** Soil microbial properties at the different slope positions.

Using Type	Slope Position	Treatment	MBC mg kg^−1^	MBN mg kg^−1^	MBP mg kg^−1^	HP mg g^−1^	SE mg g^−1^	Ur mg g^−1^	ACP mg g^−1^	ALP mg g^−1^
Citrus plots	Upper	C-CK	73.97 ± 4.32 d	16.98 ± 0.78 c	5.25 ± 0.37 d	2.50 ± 0.09 b	14.20 ± 0.67 c	3.58 ± 0.08 b	0.34 ± 0.01 d	0.63 ± 0.03 d
		WC	204.54 ± 10.26 a	25.73 ± 0.89 a	37.03 ± 0.64 b	2.07 ± 0.16 c	16.17 ± 1.28 a	2.63 ± 0.11 c	0.38 ± 0.02 b	0.66 ± 0.03 c
		SM	133.07 ± 7.67 b	20.95 ± 0.82 b	43.44 ± 2.15 a	3.27 ± 0.28 a	14.20 ± 0.63 c	3.61 ± 0.14 b	0.43 ± 0.00 a	0.84 ± 0.03 a
		HF	109.04 ± 5.78 c	18.63 ± 1.01 bc	18.28 ± 1.37 c	2.12 ± 0.07 c	14.87 ± 0.72 b	3.91 ± 0.22 a	0.36 ± 0.01 c	0.73 ± 0.02 b
	Middle	C-CK	115.55 ± 9.98 d	20.32 ± 1.22 b	16.49 ± 0.23 c	3.19 ± 0.26 d	13.83 ± 0.43 d	3.85 ± 0.21 d	0.39 ± 0.00 d	0.70 ± 0.02 c
		WC	220.79 ± 8.87 a	43.87 ± 1.76 a	51.90 ± 1.45 a	3.28 ± 0.05 c	16.48 ± 1.01 a	5.51 ± 0.07 a	0.54 ± 0.02 a	0.67 ± 0.00 d
		SM	173.12 ± 4.04 c	40.93 ± 0.64 a	49.47 ± 1.54 a	4.66 ± 0.15 a	15.58 ± 0.87 b	4.21 ± 0.11 c	0.47 ± 0.02 c	0.86 ± 0.02 a
		HF	211.56 ± 8.54 b	23.78 ± 0.74 b	35.04 ± 1.24 b	3.79 ± 0.21 b	15.31 ± 0.35 c	4.57 ± 0.15 b	0.50 ± 0.01 b	0.80 ± 0.01 b
	Lower	C-CK	242.55 ± 7.54 a	18.81 ± 1.06 c	31.91 ± 1.36 c	3.54 ± 0.11 c	17.72 ± 0.86 b	4.29 ± 0.16 d	0.42 ± 0.02 d	0.85 ± 0.05 d
		WC	265.06 ± 15.35 a	99.68 ± 1.43 a	102.21 ± 1.07 a	4.64 ± 0.13 a	19.40 ± 0.94 a	4.87 ± 0.25 c	0.52 ± 0.03 c	1.00 ± 0.02 b
		SM	263.91 ± 9.38 a	100.23 ± 2.57 a	75.08 ± 0.68 b	2.59 ± 0.06 d	17.97 ± 1.50 b	5.21 ± 0.29 b	0.57 ± 0.00 a	0.88 ± 0.01 c
		HF	264.08 ± 11.67 a	69.64 ± 0.86 b	70.38 ± 1.92 b	4.40 ± 0.19 b	20.09 ± 0.50 a	5.33 ± 0.36 a	0.53 ± 0.02 b	1.05 ± 0.03 a
Crop plots	Upper	W-CK	62.10 ± 3.49 b	18.72 ± 0.64 d	3.34 ± 0.28 c	3.21 ± 0.28 c	10.37 ± 0.12 d	2.76 ± 0.15 d	0.34 ± 0.03 d	0.60 ± 0.00 b
		TS	74.09 ± 2.58 ab	41.30 ± 1.22 a	6.57 ± 0.31 a	3.48 ± 0.22 b	18.62 ± 1.04 a	4.11 ± 0.31 a	0.43 ± 0.02 a	0.65 ± 0.02 a
		AF	73.28 ± 4.64 ab	30.37 ± 1.58 c	5.28 ± 0.23 b	3.73 ± 0.25 a	13.09 ± 0.66 c	3.49 ± 0.27 c	0.36 ± 0.01 c	0.67 ± 0.01 a
		RS	82.89 ± 3.12 a	35.14 ± 1.43 b	5.12 ± 0.36 b	3.21 ± 1.56 c	15.87 ± 0.54 b	3.73 ± 0.05 b	0.37 ± 0.00 b	0.59 ± 0.01 b
	Middle	W-CK	79.35 ± 7.35 c	35.85 ± 1.52 d	7.07 ± 0.15 c	2.38 ± 0.14 d	10.74 ± 0.58 c	3.19 ± 0.08 c	0.35 ± 0.00 c	0.73 ± 0.01 d
		TS	139.86 ± 6.55 a	99.77 ± 3.64 a	9.74 ± 0.17 b	3.79 ± 0.16 b	18.98 ± 0.82 a	5.23 ± 0.16 a	0.50 ± 0.02 a	0.79 ± 0.02 b
		AF	132.12 ± 7.63 a	89.11 ± 1.25 b	12.99 ± 0.43 a	3.99 ± 0.08 a	14.83 ± 0.43 b	5.33 ± 0.18 a	0.46 ± 0.02 b	0.76 ± 0.05 c
		RS	99.18 ± 2.97 b	46.21 ± 3.29 c	9.10 ± 0.41 b	3.57 ± 0.06 c	14.46 ± 0.35 b	4.01 ± 0.08 b	0.44 ± 0.00 b	0.81 ± 0.03 a
	Lower	W-CK	89.50 ± 5.72 c	42.67 ± 1.87 c	11.12 ± 0.58 d	3.76 ± 0.12 c	11.28 ± 0.36 d	3.58 ± 0.19 c	0.51 ± 0.01 d	0.81 ± 0.01 b
		TS	171.82 ± 6.93 a	93.89 ± 2.15 a	25.13 ± 0.92 b	4.63 ± 0.08 a	20.52 ± 0.45 a	4.78 ± 0.13 a	0.59 ± 0.04 a	0.82 ± 0.01 b
		AF	160.04 ± 2.49 a	94.25 ± 3.61 a	29.98 ± 1.38 a	4.10 ± 3.14 b	19.37 ± 0.69 b	4.69 ± 0.10 a	0.56 ± 0.01 b	1.01 ± 0.03 a
		RS	108.45 ± 6.51 b	76.21 ± 1.16 b	18.45 ± 0.49 c	4.00 ± 1.34 b	17.79 ± 0.81 c	4.40 ± 0.12 b	0.53 ± 0.01 c	0.83 ± 0.02 b

Notes: Data represent the mean values ± standard errors (*n* = 3). Different letters indicate significant differences at *p* < 0.05. MBC = microbial biomass carbon; MBN = microbial biomass nitrogen; MBP = microbial biomass phosphorus; HP = hydrogen peroxidase; SE = sucrose enzyme; Ur = urease; ACP = acid phosphatase; ALP = alkaline phosphatase.

**Table 2:** Discriminant analysis of the influence of slope position and treatment on soil properties.

Classification		Discriminant Function	Eigenvalue	% of Variance	Canonical Correlation	Wilk’s Lambda	*p*-Value
Citrus plots	Treatments	1	11,400.527	99.4	1.000	0.000	0.000
		2	42.265	0.4	0.988	0.001	0.000
		3	28.501	0.2	0.983	0.034	0.002
	Slope positions	1	688.797	99.5	0.999	0.000	0.000
		2	3.456	0.5	0.881	0.224	0.084
Crop plots	Treatments	1	9.400	100.0	0.951	0.096	0.000
	Slope positions	1	3.449	100.0	0.880	0.225	0.001

**Table 3:** Annual fertilization amount in the field experiment (kg ha^−1^).

Use Type		Time	N	P_2_O_5_	K_2_O
Citrus plots		Early March	88	36	36
		Late May	198	81	81
		Mid-November	154	63	63
Crop plots	Peanut/Sesame	Early June	176	80	96
	Wheat/Ryegrass	Mid-October	135	60	75

**Table 4:** Methods for determining soil properties.

Properties	Units	Methods	References
Soil particle size	%	Laser diffraction method	[55]
Hydrogen ion concentration (pH)	-	Potentiometric method	[56]
Cation exchange capacity (CEC)	cmol kg^−1^	Ammonium acetate method	[57]
Soil organic matter (SOM)	g kg^−1^	Dichromate method	[57]
Total nitrogen (TN)		Kjeldahl digestion procedure	[58]
Total phosphorus (TP)		Sodium carbonate fusion method	[59]
Total potassium (TK)		Sodium hydroxide fusion method	[60]
Available phosphorus (AP)		Molybdenum antimony anticolorimetric method	[54]
Available potassium (AK)		Flame photometer method	
Total Fe (TFe)		Inductively coupled plasma atomic emission spectroscopy	[61]
Total boron (TB)		Azomethine H colorimetric method	[62]
Alkaline phosphatase (ALP)	mg g^−1^	Disodium phenyl phosphate colorimetric method	[63]
Acid phosphatase (ACP)		Disodium phenyl phosphate colorimetric method	
Urease (Ur)		Phenol-sodium hypochlorite colorimetric method	
Hydrogen peroxidase (HP)		KMnO_4_ titration method	
Sucrose enzyme (SE)		3,5-Dinitrosalicylic acid colorimetric method	
Microbial biomass carbon (MBC)		Chloroform fumigation incubation method	[54]
Microbial biomass nitrogen (MBN)		The same as above	
Microbial biomass phosphorus (MBP)		The same as above	

## Data Availability

Data are contained within the article.

## Abbreviations

CAs, conservation agriculture practices; TGRA, Three Gorges Reservoir area; SQI, soil quality index; C-CK, conventional citrus planting pattern; WC, citrus intercropped with white clover; SM, citrus orchard soil mulched with straw; HF, citrus intercropped with *Hemerocallis flava* contour hedgerows; W-CK, conventional wheat–peanut rotation; TS, wheat–peanut rotation intercropped with *Toona sinensis* contour hedgerows; AF, wheat–peanut rotation intercropped with alfalfa contour hedgerows; RS, ryegrass–sesame rotation.
